# Metabolic Engineering for Unusual Lipid Production in *Yarrowia lipolytica*

**DOI:** 10.3390/microorganisms8121937

**Published:** 2020-12-06

**Authors:** Young-Kyoung Park, Jean-Marc Nicaud

**Affiliations:** Micalis Institute, AgroParisTech, INRAE, Université Paris-Saclay, 78352 Jouy-en-Josas, France; jean-marc.nicaud@inrae.fr

**Keywords:** *Yarrowia lipolytica*, oleochemicals, lipids, unusual lipids, metabolic engineering

## Abstract

Using microorganisms as lipid-production factories holds promise as an alternative method for generating petroleum-based chemicals. The non-conventional yeast *Yarrowia lipolytica* is an excellent microbial chassis; for example, it can accumulate high levels of lipids and use a broad range of substrates. Furthermore, it is a species for which an array of efficient genetic engineering tools is available. To date, extensive work has been done to metabolically engineer *Y. lipolytica* to produce usual and unusual lipids. Unusual lipids are scarce in nature but have several useful applications. As a result, they are increasingly becoming the targets of metabolic engineering. Unusual lipids have distinct structures; they can be generated by engineering endogenous lipid synthesis or by introducing heterologous enzymes to alter the functional groups of fatty acids. In this review, we describe current metabolic engineering strategies for improving lipid production and highlight recent researches on unusual lipid production in *Y. lipolytica*.

## 1. Introduction

Microbial lipids are promising alternative fuel sources given growing concerns about climate change and environmental pollution [1,2,3,4]. They offer multiple advantages over plant oils and animal fats. For example, the production of microbial lipids does not result in resource competition with food production systems; is largely independent of environmental conditions; can be based on diverse substrates; and allows product composition to be customized based on the desired application [3,4]. As a result of recent advances in metabolic engineering and synthetic biology, it is now possible to produce a wide range of oleochemicals in both bacteria and yeast.

Unusual lipids differ from usual lipids in chain length (short, very long, and odd numbered), the position or number of double bonds (conjugated and polyunsaturated), and/or functional group type (e.g., hydroxy, epoxy, keto, branched chain, and cyclic) [5,6]. Unusual lipids can be highly valuable because they have applications in the pharmaceutical, food, and chemical industries. However, their low abundance in nature makes their mass production difficult. Consequently, using microbes to produce unusual lipids may serve as an environmentally sustainable and economically viable alternative [3,7,8]; for example, such lipids are naturally generated in a more finalized form because the need for chemical processes is reduced or avoided during manufacturing [3].

When it comes to the production of oleochemicals, oleaginous chassis display the clear advantage of accumulating larger quantities of lipids (>20% of dry cell weight (DCW)). *Yarrowia lipolytica*, a well-studied oleaginous yeast, is considered to be a promising host because it utilizes a broad range of substrates, has efficient engineering toolkits, and is easy to cultivate at large scales [3,9]. Consequently, numerous studies have used metabolic engineering and the optimization of cultivation conditions to increase lipid titers, yield, and productivity in *Y. lipolytica*. To date, the focus has mainly been on usual lipids. Strains have been successfully engineered to produce more than 80 g/L of lipids from a range of substrates [10,11,12]. More recently, interest has grown in producing unusual lipids, which are of high industrial value. This trend has been accompanied by the development of new synthetic biology tools and gene editing technology for *Y. lipolytica* [13,14]. Here, we review recent advances in the production of unusual lipids via metabolic engineering in this non-conventional yeast. We also briefly summarize the general dynamics of lipid metabolism and engineering strategies for lipid production in *Y. lipolytica*. Finally, we describe different approaches for producing specific unusual lipids, such as engineering the endogenous lipid synthesis system or introducing heterologous enzymes.

## 2. Lipid Production in *Yarrowia lipolytica*

**Figure 1:**
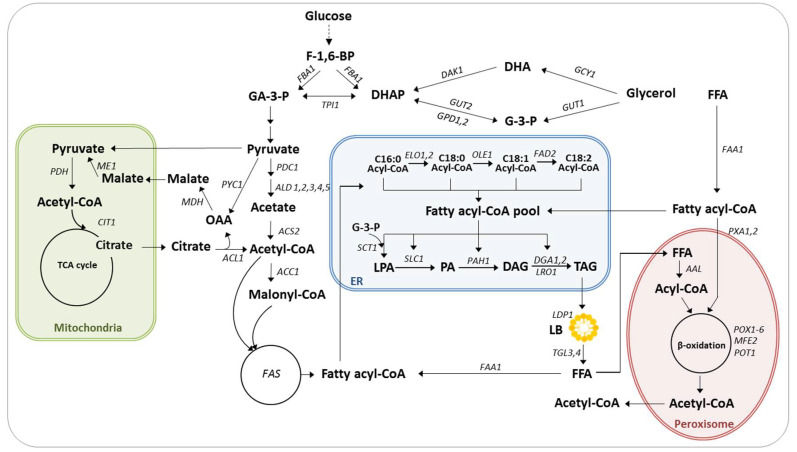
Lipid metabolism in *Y. lipolytica*. Abbreviations: F-1,6-BP, fructose-1,6-biphosphate; GA-3-P, glyceraldehyde-3-phosphate; DHAP, dihydroxyacetone phosphate; DHA, dihydroxyacetone; G-3-P, glycerol-3-phosphate; OAA, oxaloacetate; FFA, free fatty acid; LPA, lysophosphatidic acid; PA, phosphatidic acid; DAG, diacylglycerol; TAG, triacylglycerol; ER, endoplasmic reticulum; LB, lipid body; *FBA1*, fructose biphosphate aldolase; *TPI1*, triosephosphate isomerase; *ME1*, malic enzyme; *PYC1*, pyruvate carboxylase; *MDH*, malate dehydrogenase; *PDH*, pyruvate dehydrogenase; *CIT1*, citrate synthase; *CTP1*, citrate/malate transporter; *PDC1*, pyruvate decarboxylase; *ALD1-5*, aldehyde dehydrogenase; *ACS2*, acetyl-CoA synthetase; *ACC1*, acetyl-CoA carboxylase; FAS, fatty acid synthase; *FAA1*, fatty acyl-CoA synthetase; *GPD1* and *GPD2*, glycerol-3-phosphate dehydrogenase; *GUT1* and *GUT2*, glycerol kinase; *DAK1*, dihydroxyacetone kinase; *GCY1*, glycerol dehydrogenase; *ELO1, 2*, elongase; *OLE1*, ∆-9 desaturase; *FAD2*, ∆-12 desaturase; *SCT1*, glycerol-3-phosphate O-acyltransferase; *SLC1*, fatty acyl transferase; *PAH1*, phosphatidate phosphatase; *DGA1, 2*, DAG acyltransferase; *LRO1*, phospholipid:diacylglycerol acyltransferase; *LDP1*, lipid droplet protein; *TGL3, 4*, TAG lipase 3 and 4; *AAL*, acyl/aryl-CoA ligase; *POX1-6*, acyl-CoA oxidases 1-6; *MFE2*, peroxisomal multifunctional enzyme 2; *POT1*, 3-ketoacyl-CoA thiolase; *LIP2,7*, and *8*, lipase; *PXA1, 2*, peroxisomal ATP-binding cassette transporter complex; dashed lines, multistep metabolic route.

*Y. lipolytica* is a well-characterized oleaginous yeast that displays a high flux of acetyl-CoA and an oil sequestration mechanism. The species’ lipid metabolism has been thoroughly studied (Figure 1), and its lipid accumulation has been enhanced via engineering.

### 2.1. Lipid Metabolism

De novo fatty acid (FA) synthesis occurs in the cytosol. It begins with acetyl-CoA, which can arise from reactions mediated by acetyl-CoA synthetase (ACS, encoded by *YALI0F05962g*), pyruvate dehydrogenase complex (PDH), or ATP citrate lyase (ACL, encoded by *YALI0E34793g* and *YALI0D24431g*) [15]. Interestingly, *ACL* genes are only present in the genomes of oleaginous yeast, as they are a hallmark of oleaginous microorganisms [16]. This acetyl-CoA is then converted into malonyl-CoA by acetyl-CoA carboxylase (Acc1, encoded by *YALI0C11407g*).

The fatty acid synthase (FAS) enzymatic complex (encoded by *YALI0B15059g* and *YALI0B19382g*) produces acyl-CoA by adding two carbons; acetyl-CoA serves as the initiation molecule, and malonyl-CoA serves as the elongation unit. The acyl-CoA molecules with chain length of 16 or 18 carbons are then transported into the endoplasmic reticulum (ER) for further elongation and desaturation by elongases (Elo1p encoded by *YALI0F06754g* and Elo2p encoded by *YALI0B20196g*) and desaturases (Ole1p; Δ9 desaturase encoded by *YALI0C05951g* and Δ12 desaturase; Fad2p encoded by *YALI0B10153g*) [3,17,18].

The acyl-CoA products are part of a condensation reaction mediated by glycerol-3-phosphate (G3P). This process generates lysophosphatidic acid (LPA) via the action of G3P acyltransferase (Sct1p, encoded by *YALI0C00209g*), then phosphatidic acid (PA) via the action of LPA acyltransferase (Slc1p, encoded by *YALI0E18964g*), and finally diacylglycerol (DAG) via the action of phosphatidate phosphatase (Pah1p, encoded by *YALI0D27016g*). In the last step, DAG is converted into triacylglycerol (TAG) via the Kennedy pathway. This conversion process either involves phospholipids and the action of phospholipid:diacylglycerol acyltransferase (Lro1p, encoded by *YALI0E16797g*) or acyl-CoA and the action of DAG acyltranferases (Dga1p and Dga2p, encoded by *YALI0E32769g* and *YALI0D07986g*, respectively) [19,20].

*Y. lipolytica* and other oleaginous yeasts mostly store their lipids as TAGs (80–90% of the neutral lipid fraction); a smaller quantity are stored as steryl esters (SEs) in lipid bodies (LBs). Stored lipids are used as additional carbon sources under nutrient-limited environment through remobilization, transport, and degradation. TAGs are hydrolyzed to free fatty acids (FFAs) by two lipases: Tgl4p (encoded by *YALI0F10010g*), an active lipase found at the LB interface, and Tgl3p (encoded by *YALI0D17534g*), which is a positive regulator of Tgl4p [21]. FFAs are then activated and transported to peroxisomes to be degraded. There are two possible routes: (1) FFAs can be activated by Faa1p in the cytoplasm and then transported to the peroxisome by the transporters Pxa1p/Pxa2p (encoded by *YALI0A06655g*/*YALI0D04246g*) or (2) they can be transported to the peroxisome and then activated by acyl/aryl-CoA-ligases (AALs) which required ATP provided by the ATP transporter Ant1p (encoded by *YALI0E03058g*) [22,23].

FA degradation takes place in the peroxisome via the β-oxidation pathway, which is a four-reaction cycle that shortens the FA backbone by two carbons and releases acetyl-CoA. In *Y. lipolytica*, the first step is carried out by six acyl-CoA oxidases encoded by *POX* genes (*YALI0E32835g*, *YALI0F10857g*, *YALI0D24750g*, *YALI0E27654g*, *YALI0C23859g*, and *YALI0E06567g*); these enzymes display different activity patterns depending on FFA chain length [24]. The second and third steps in β-oxidation are catalyzed by Mfe2p (a multifunctional enzyme encoded by *YALI0E15378g*). The fourth and last step in β-oxidation is carried out by Pot1p (a thiolase encoded by *YALI0E18568g*).

### 2.2. Metabolic Engineering to Improve Lipid Production

#### 2.2.1. Increasing the Size of Precursor Pools

A common starting point in metabolic engineering is increasing the supply of precursors. In the case of FAs, these precursors are acetyl-CoA and malonyl-CoA. *Y. lipolytica* has the natural ability to produce high levels of cytosolic acetyl-CoA because of the yeast’s ACL activity (Acl1p, Acl2p), which is particularly strong under conditions of nitrogen limitation. An attempt was made to decouple acetyl-CoA flux from nitrogen starvation by engineering several alternative cytosolic acetyl-CoA pathways, including the pyruvate-acetate route (mediated by pyruvate decarboxylase *pdc*, aldehyde dehydrogenase *aldH*, and acetyl-CoA synthase *acs*); the pyruvate-aldehyde route (mediated by pyruvate decarboxylase *pdc* and CoA-acetylating aldehyde dehydrogenase *aad*); the pyruvate formate lyase (*pflA* and *pflB*) and acetyl-CoA shuttling pathway (mediated by carnitine acetyltransferase *cat2*); and the non-oxidative pentose-phosphate pathway (mediated by phosphoketolase (PK) and phosphotransacetylase *pta*) [25]. The overexpression of native *ACL* only slightly increased lipid titers, as seen elsewhere [26]. Most of the other strategies, however, led to significant improvements. In particular, lipid titers improved by 75% when there was overexpression of the peroxisomal carnitine acetyltransferase (Cat2) taken from *Saccharomyces cerevisiae*, which enhanced the export of mitochondrial acetyl-CoA into the cytosol. Moreover, the engineered strain began accumulating lipids during the exponential growth phase before nitrogen became limiting. Using fed-batch cultivation, high lipid levels (66.4 g/L) and oil content (81.4%, g/g DCW) were achieved, with an overall yield of 0.229 g/g of glucose.

Several studies have shown that *ACC1* overexpression increases lipid accumulation by providing malonyl-CoA [25,27,28]. This strategy can increase lipid content two-fold, resulting in a greater representation of linoleic acid among total lipids [27]. In the same study, co-expression of *ACC1* and *DGA1* (the gene encoding DAG acyltransferase) significantly increased lipid content 4.7- and 2.3-fold compared to the control and the *ACC1*-overexpressing strain, respectively. In a bioreactor, the strain overexpressing *ACC1* and *DGA1* had a lipid content of 61.7% (g/g DCW), and its overall yield and productivity from glucose were 0.195 g/g and 0.143 g/L/h, respectively. This simple but efficient method has shown that the push-and-pull strategy is extremely successful in enhancing lipid production.

The same group sought to overcome the potential allosteric inhibition of Acc1p by saturated FAs. To this end, they overexpressed delta-9 stearoyl-CoA desaturase (SCD, encoded by *YALI0C05951g*) to convert saturated FAs into monounsaturated FAs. The engineered strain had higher lipid titers (55 g/L from glucose), oil content (67% g/g DCW), overall yield (0.234 g/g), and productivity (0.707 g/L/h) [28].

#### 2.2.2. Increasing Lipogenic Metabolic Flux

Several studies have shown that diverting the carbon flux to TAGs can improve lipid production. The push-and-pull strategy described above (i.e., in which *ACC1* and *DGA1* are co-expressed) provides a good example of this approach [27]. Research has shown that Dga1p and Dga2p play an important role in boosting lipogenesis because they catalyze the last step in TAG synthesis [19,27,29,30]. The overexpression of these enzymes has successfully improved lipid production by influencing LB phenotype—*DGA1* overexpression generates smaller but more numerous LBs, and *DGA2* overexpression results in the formation of larger LBs [30]. Consequently, the strategy of enhancing the last step of TAG synthesis has generally been coupled with other engineering strategies.

Heterologous expression of *DGA1* and *DGA2* in *Y. lipolytica* has also been successfully used to engineer a lipid-overproducing phenotype. For instance, when *DGA1* from *Rhodosporidium toruloides* and *DGA2* from *Claviceps purpurea* were expressed in *Y. lipolytica*, they resulted in better performance than when their native equivalents were expressed [12]. The overexpression of these heterologous genes combined with the deletion of *TGL3*, which encodes the regulatory protein involved in TAG remobilization, resulted in a lipid content level of 77% (g/g DCW) and a yield of 0.21 g/g during batch cultivation. When a fed-batch process with glucose was used, the strain produced 85 g/L of lipids and had a productivity level of 0.73 g/L/h.

Another strategy can successfully improve lipid accumulation by redirecting carbon flux [31]. Because the G3P shuttle pathway provides the TAG backbone, it is closely tied to TAG synthesis. When G3P concentrations increase because of the overexpression of *GPD1* and/or the deletion of *GUT2*, TAG synthesis also increases. Further engineering that couples β-oxidation inactivation and *GPD1* overexpression leads to tandem increases in G3P concentrations and FA availability, resulting in more pronounced lipid accumulation (up to 65% g/g DCW).

#### 2.2.3. Inhibiting Lipid Remobilization and Degradation

TAGs stored in LBs are transported to the peroxisome and degraded by β-oxidation. These processes are part of an antagonistic pathway leading to lipid synthesis and accumulation. Therefore, the genes involved in lipid transport and degradation are useful deletion targets in efforts to improve lipid production.

It is known that intracellular lipases encoded by *TGL3* and *TGL4* are involved in TAG remobilization. In *Y. lipolytica*, the inactivation of Tgl3p and/or Tgl4p led to higher levels of lipid accumulation—amounts of TAGs and FFAs increased, but FA profiles were unaffected [21]; furthermore, Tgl4p was found to be a major intracellular lipase that was activated by Tgl3p. Lipid accumulation was boosted by both deleting *TGL3* and overexpressing heterologous *DGA1* and *DGA2* in *Y. lipolytica* [12].

When the β-oxidation pathway was inhibited via the inactivation of *POX* genes, greater lipid accumulation occurred. The deletion of the *POX1-6* genes combined with *GUT2* deletion resulted in a 3.2-fold increase in lipid content (from 12.76% to 41.92%, g/g DCW); the engineered strain exhibited a hyper lipid-accumulating phenotype with extremely large LBs [32]. When β-oxidation was knocked out and both *DGA2* and *GPD1* were overexpressed, lipid accumulation levels reached 55% (g/g DCW) under nitrogen-limited conditions [33,34].

It has been shown that inactivating MFE, which catalyzes the second and the third step in β-oxidation, can also improve lipid accumulation. Moreover, the deletion of *GUT2*, *POX1-6*, and *MFE1* combined with the overexpression of *GPD1* was also found to drive higher levels of lipid accumulation (65–75% g/g DCW) [31].

Another strategy for increasing lipid accumulation has been to allow β-oxidation but to disrupt peroxisome biogenesis, notably by deleting the genes *PEX3*, *PEX10*, and *PEX11* [35,36]. A 60-fold increase in lipid titers (25 g/L) and a lipid content of nearly 90% (g/g DCW) were obtained by combining the deletion of *PEX10* and *MFE2* with the overexpression of *DGA1* [29]. This engineered strain was used for an evolutionary approach with a floating cell enrichment process, enhanced lipid titers of up to 39.1 g/L and a lipid content of 77.6% (g/g DCW) were achieved [37].

#### 2.2.4. Engineering Redox Metabolism

In lipid synthesis, a crucial variable is the availability of the FAS cofactor, NADPH, which converts acetyl groups into fully reduced acyl chains. Traditionally, the cytosolic NADP^+^-dependent malic enzyme is thought to be the major producer of lipogenic NADPH and to operate via a trans hydrogenation mechanism termed the pyruvate-oxaloacetate-malate (POM) cycle [38,39]. However, in *Y. lipolytica*, a malic enzyme is predicted to be found in the mitochondria, which is inconsistent with what is known for other oleaginous yeasts. Further, in the same species, the overexpression of the native malic enzyme did not increase lipid production, and the enzyme had a greater affinity for NAD^+^ than for NADP^+^ [16,40].

Many studies have suggested that *Y. lipolytica* regenerates NADPH almost exclusively through the oxidative pentose phosphate pathway (oxPPP) [41]. As determined by ^13^C metabolic flux analysis, flux through the oxPPP was significantly enhanced in an engineered strain overexpressing *DGA1* and *ACC1*. Furthermore, the estimated rate of NADPH synthesis via the oxPPP was consistent with the estimated rate of NADPH consumption in the TAG synthesis pathway in engineered strains. This idea has received additional support from research in which the overexpression of related enzymes enhanced the flux through the oxPPP and increased lipid synthesis [42,43,44]. For example, Dobrowolsk and Mirończuk investigated each gene of the PPP, the overexpression of transketolase (*TKL1*, *YALI0E06479g*) in DGA1-overexpressed strain showed the increase of lipid content and titer (23.94% (g/g DCW), 1.42 g/L) over the control strain (16.89% (g/g DCW), 0.83 g/L) with glycerol as a sole carbon source [44]. In another study, an analysis of the metabolic network during lipid production revealed an imbalance in electron cofactors—NADH was present in excess, whereas NADPH was limiting [11]. To convert the excess NADH into NADPH and thus enhance lipid production, several pathway modules were constructed [11]. Two NADP^+^-dependent glyceraldehyde-3-phosphate dehydrogenase genes (*GapC* from *Clostridium acetobutylicum* and *GPD1* from *Kluyveromyces lactis*) were overexpressed in a strain with an *ACC1*- and *DGA1*-overexpressing background; lipid accumulation improved by 20% and 17.8%, respectively. Research was also performed to determine whether a cytosolic NADP^+^-dependent malic enzyme, Mce2p from *Mucor circinelloides*, could activate the POM cycle and further boost the conversion of NADH to NADPH. The introduction of Mce2p improved yield by 23%. When GapC introduction was coupled with *YEF* (endogenous NAD^+^/NADH kinase) overexpression, the result was multiple functional synthetic pathways working simultaneously to convert NADH to NADPH. In the final engineered strain, lipid titers, productivity, and yield reached 99 g/L, 1.2 g/L/h, and 0.27 g/g, respectively. 

#### 2.2.5. Removing Competing Byproducts

In addition to TAG, glycogen can also store excess carbon. Glycogen starts to accumulate only after the complete depletion of the nitrogen source, which is the same pattern as seen with TAG accumulation. Therefore, research examined whether redirecting carbon flux from glycogen synthesis to TAG synthesis could improve lipid accumulation [45]. It was found that glycogen could account for up to 16% of biomass in wild-type (WT) *Y. lipolytica* (strain W29). In a strain in which the gene for glycogen synthase (Gsy1p, encoded by *YALI0F18502g*) had been deleted, TAG accumulation was 60% higher than in the WT strain. TAG accumulation increased even further, from 44.9% to 52.4% (g/g DCW), when *TGL4* was also deleted and *DGA2* and *GPD1* were overexpressed.

## 3. Unusual Lipids: Fatty Acids with Tailored Chain Lengths 

One of the advantages of microbial lipid production is that lipid profiles can be customized to suit industrial applications. Lipids with unusual chain lengths are increasingly the target of metabolic engineering because they are rare in nature, they have numerous applications, and they are economically valuable.

The TAG oil naturally produced by *Y. lipolytica* is mainly composed of long-chain FAs, palmitic acid (C16:0), palmitoleic acid (C16:1 Δ9), stearic acid (C18:0), oleic acid (C18:1 Δ9), and linoleic acid (C18:2 Δ9Δ12). Studies that have successfully synthesized FAs with specific chain lengths, such as medium-chain FAs (MCFAs), odd-chain FAs (OCFAs), and very-long-chain FAs (VLCFAs) are described in this section and in Table 1.

### 3.1. Medium-Chain Fatty Acids

MCFAs (chain length range: C6–C12) are valuable compounds in the chemical industry. They can be used as biofuels as well as intermediate fine chemicals for generating plastics, surfactants, biocontrol agents, and cosmetics [46,47]. Since MCFAs are present in very low concentrations in only a few seed oils (i.e., coconut and palm kernel oils), an alternative source of MCFAs could be microbial production utilizing renewable biomass, which would be a more environmentally sustainable and economically feasible approach [47,48]. Because FA chain length is associated with the termination of the elongation cycle during FA synthesis, research has been performed on thioesterases that have a specificity for MCFAs [49,50]. Furthermore, biochemical and structural analyses have been carried out to characterize the FAS elongation mechanism with a view to modifying FA chain length [51,52,53].

In *Y. lipolytica*, the heterologous acyl-ACP thioesterases (ACPTs) from plants and bacteria can terminate elongation early to produce MCFAs, namely decanoic acid (C10-FA) or octanoic acid (C8-FA) [54]. Among the five heterologous ACPTs tested, four resulted in the production of C10-FA (representing 36–57% of total lipids). The strain expressing ACPT from *Umbellularia californica* produced the highest ratio of C8-FA (14% of total lipids). The MCFAs produced were found to represent all the different lipid classes, including TAGs, DAGs, MAGs, sterols, steryl esters, and FFAs. This finding indicates that the enzymes were successfully incorporated into the native machinery. Plant, fungal, and bacterial fatty ACPTs were also screened to determine whether they displayed distinct chain-length specificities when producing MCFAs [25]. In *Y. lipolytica*, the expression of *fatB2* from *Cocos nucifera* resulted in the production of C12-FA (2.3% of total lipids), and the expression of *ybgC* from *Escherichia coli* resulted in the production of C14-FA (up to 19.2% of total lipids). An attempt was made to further increase MCFA levels by introducing the bifunctional acyl-ACP/acyl-CoA thioesterase from *E. coli* (EcTesA). To bridge the gap in substrate specificity between native FAS and thioesterase, the malonyl/palmitoyl transacylase (MPT) domain of the native FASI was substituted with a truncated FAS1 fused with thioesterases (hFAS-TEs). The hybrid hFAS-TEs increased C12-FA and C14-FA production. For example, the strain expressing hFAS-EcTesA’ (truncated EcTesA) displayed a boost in C14-FA levels (up to 29.2% of total lipids).

A mutation was introduced into the native FAS system of *Y. lipolytica* to determine the effects on MCFA production [55]. It was hypothesized that ketoacyl synthase (the KS domain in Fas1p) catalyzes the condensation reaction between acyl-ACP and malonyl-CoA and thus determines chain length during the production of long-chain FAs (C16 and C18). The FA binding site (Ile1220) in the KS domain, as predicted via molecular modeling, was replaced with various amino acid residues. MCFAs (mostly C14-FA) were produced when the binding site was replaced by an aromatic amino acid. The best performing mutant, I1220W, accumulated C14-FA at levels representing 11.6% in total FAs. The I1220W strain underwent additional engineering to further improve C14-FA accumulation [56]: to inhibit the elongation of C14-FA and C16-FA, *ELO1* was deleted. As a result, C14-FA was the predominant compound produced by the mutant (87% of total lipids). Heterologous diglyceride acyltransferase (DGAT) from *Elaeis guineensus* was also introduced to boost the accumulation of MCFAs in the form of TAGs. C14-FA content reached 1.25 g/L, which is 1.7 times higher than the amount seen in the control strain.

### 3.2. Odd-Chain Fatty Acids

OCFAs represent less than 3% of the total FAs naturally synthesized by microorganisms, plants, and animals. However, they have various important industrial applications. For example, *cis*-9-heptadecenoic acid (C17:1) has anti-inflammatory properties and can help treat psoriasis, allergies, and autoimmune diseases when used in pharmaceuticals or cosmetics [57]. In the chemical industry, *cis*-9-heptadecenoic acid is used as a biocontrol agent in the fight against powdery mildew, a fungal disease that affects a wide range of plants [58]. In addition, OCFAs and their derivatives are precursors for manufacturing flavor and fragrance compounds, hydraulic fluids, plasticizers, coatings, and other industrial substances [58,59,60,61].

The synthesis of OCFAs diverges from that of ECFAs during the first cycle of FA elongation: propionyl-CoA merges with malonyl-CoA instead of with acetyl-CoA [62]. To provide a source of propionyl-CoA, it is common to utilize compounds with three-carbon chains, such as propionate, propionic acid, or 1-propanol.

Previous work in *E. coli* found that OCFA production could be improved via metabolic engineering strategies and propionate supplementation [63]. One strategy was to introduce the propionyl-CoA synthetase gene (*SeprpE*) from *Salmonella enterica* to increase intracellular propionyl-CoA availability resulting from propionate supplementation. Another was to switch out the native β-ketoacyl-ACP synthase III (encoded by *fabH*) with BsFabH1 from *Bacillus subtilis* to enhance propionyl-CoA specificity. The final strain overexpressed *SeprpE* and *SafabH* and produced 1.205 g/L of OCFAs (60.25% of total lipids), with C15-FA predominating.

Several studies have shown that, in *Y. lipolytica*, the WT strain can use propionate to produce high levels of OCFAs (up to 43% of total lipids) [64,65]. When propionyl-CoA catabolism was inhibited, the representation of OCFAs among total lipids climbed from 28.3% to 46.8% [66]. *Y. lipolytica* was further engineered (*mfe1Δ tgl4Δ pTEF-GPD1 pTEF-DGA2*) to accumulate large amounts of lipids, increasing both OCFA levels and total lipid content; the final strain produced 0.75 g/L of OCFAs, mainly C17:1-FA, thanks to an optimized fed-batch co-feeding strategy [66]. This strain was further modified to boost propionyl-CoA pools by screening for propionate-activating enzymes [67]. The highest levels of OCFA production (3.8 times higher than those in the control strain) were obtained by expressing propionyl-CoA transferase (PCT) from *Ralstonia eutropha*. To further increase OCFA production, pools of β-ketovaleryl-CoA were bolstered by introducing β-ketothiolase (BktB) from *R. eutropha*. The final strain produced 1.87 g/L of OCFAs, which accounted for 62% of total lipids (C/N ratio = 45).

Glucose has also been used to drive the endogenous synthesis of propionyl-CoA and OCFA production in *Y. lipolytica* [68]. Overexpression of the aspartate/α-ketobutyrate pathway increased the representation of OCFAs among total lipids from 0.84% to 3.86%. When this strategy was combined with the high lipid-accumulation strategy, glucose-based OCFA production improved 7.2-fold, reaching 0.36 g/L when flask cultivation was used.

### 3.3. Very-Long-Chain Fatty Acids

Very-long-chain fatty acids (VLCFAs; C22–C26) are essential biological compounds that occur in small quantities among cellular lipids. VLCFAs are intermediate molecules in reactions that generate very-long-chain fatty alcohols (e.g., docosanol) and very-long-chain fatty waxes (e.g., Jojoba oil), which are widely used as lubricants, detergents, polymers, developing agents for photographic film, cosmetics, and pharmaceuticals [69,70,71,72,73].

Elongases that act on FA chain lengths have become targets in efforts to increase VLCFA production because they display different substrate specificity depending on their origin. For example, in *S. cerevisiae*, the endogenous FA elongation system (scELO1, scELO2, and scELO3) was engineered and the FASI system from *Mycobacterium vaccae* was introduced to enhance the production of VLCFAs ranging in chain length from C22 to C26 [72].

In *Y. lipolytica*, C20–C22 VLCFAs were synthesized by overexpressing β-ketoacyl-CoA synthases (KCSs) from *Arabidopsis thaliana* that convert C16 and C18 LCFAs into VLCFAs [74]. VLCFA production was increased three-fold by deleting the peroxisome biogenic gene, *PEX10*, and thus inhibiting LCFA-CoA catabolism. The strain was further engineered by introducing the C16/C18-elongase from *Mortierella alpina* (MaELO3) and KCS from *Crambe abyssinica*, which boosted levels of C22–24 VLCFAs. To increase quantities of acetyl-CoA, a key FA precursor, aldehyde dehydrogenase (ALDH) from *E. coli* was overexpressed; the result was a 1.7-fold increase in VLCFA titers (280 mg/L). The strain was also engineered to produce VLCFA derivatives, fatty alcohols, and wax esters by screening for heterologous enzymes—FA reductases (FARs) and wax ester synthases (WSs).

Erucic acid (C22:1 Δ13) is a VLCFA with a variety of uses. For example, it can be employed to create lubricants, surfactants, and biodiesels. In nature, it is produced by a small number of plants. However, a *Y. lipolytica* strain was recently engineered to generate erucic acid [73]. The process involved inhibiting the desaturation of C18:1 by deleting *FAD2* (Δ12 desaturase), which improved the availability of erucic acid’s precursor (C18:1). Additionally, *FAE1* from *Thlaspi arvense* (*TaFAE1*) was overexpressed to synthesize VLCFAs (C20–C24). In the engineered strain, levels of erucic acid increased to represent 6% of total FAs; levels of arachidic acid (C20:0) and gondoic acid (C20:1 Δ11) also significantly increased. VLCFA production dynamics were compared for different concentrations of two carbon sources, glycerol and waste cooking oil. For the final strain, yield was the greatest (887 mg/L) when using waste cooking oil (6% *w*/*v*).

## 4. Unusual Lipids: Fatty Acid Derivatives

**Figure 2:**
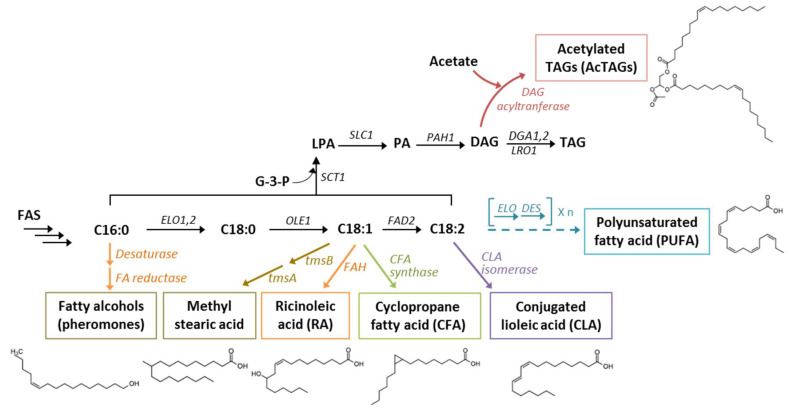
Fatty Acid (FA) derivatives that can be produced in *Y. lipolytica*. FA derivatives of interest are enclosed in colored boxes, and heterologous reactions are indicated with colored arrows. Abbreviations: AcTAGs, acetylated TAGs; FA, fatty acid; RA, ricinoleic acid; CFA, cyclopropane fatty acid; CLA, conjugated linoleic acid; FAS, fatty acid synthase; *ELO1,2*, elongase 1,2; *OLE1*, Δ9 desaturase; *FAD2*, Δ12 desaturase; *SCT1*, glycerol-3-phosphate O-acyltransferase; *SLC1*, fatty acyl transferase; *PAH1*, phosphatidate phosphatase; *DGA1,2*, DAG acyltransferase; *LRO1*, phospholipid:diacylglycerol acyltransferase; tmsB, methyltransferase; tmsA, reductase; *FAH*, ∆12-oleate hydroxylase; *ELO*, elongase; *DES*, desaturase.

Both FAs and their derivatives are compounds of interest for biotechnological applications. Depending on the enzymes introduced, a broad range of FA derivatives can be generated. For example, FFAs can be created with thioesterase, FA ethyl esters (FAEEs) with ester synthase, FA methyl esters (FAMEs) with methyl transferase, and fatty alcohols with FA reductase, just to cite a few possibilities. Some attractive FA derivatives that can be produced in *Y. lipolytica* are discussed in this section and illustrated in Figure 2.

### 4.1. Conjugated Linoleic Acids

Conjugated linoleic acids (CLAs) are the isomers of linoleic acid (C18:2, *n* = 9, 12). CLAs can be used to prevent metabolic diseases and cancer, combat atherogenesis and obesity, and modulate the immune system [75]. Consequently, the production of CLAs is of interest to the food and pharmaceutical industries. Attempts have been made to generate recombinant CLAs in *Lactobacillus planetarium, Delacroixia coronate*, *S. cerevisiae*, and various plant species [76,77,78].

In *Y. lipolytica*, an increase in CLA production (5.9% of total lipids) was obtained by expressing a large number of gene copies encoding CLA-producing isomerase from *Propionibacterium acnes* (PAI) [79]. For the best-performing strain, the rate for converting linoleic acid (LA) to CLAs was 80% under biotransformation conditions. The strain was further engineered to co-express CLA-producing isomerase and *FAD2* from *Mortierella alpine* from multiple gene copies, such that CLAs represented up to 0.4% of DCW and 10% of total FAs [80]. When grown with soybean oil-based media, the strain’s CLA production ramped up dramatically (30% of DCW and 44% of total FAs). CLA titers reached 4 g/L, including the 0.9 g/L in the growth medium. 

Another metabolic engineering approach was recently used to boost CLA production in *Y. lipolytica* [81]. It focused on blocking β-oxidation and TAG storage and overexpressing native *FAD2* and the CLA-producing isomerase (PAI) from *P. acnes*. When flask cultivation was employed, CLAs represented up to 6.5% of total FAs; when bioreactor was used, CLA titers reached 302 mg/L. Other research successfully produced *trans*-10-*cis*-12-CLA in *Y. lipolytica* by introducing *FAD2* from *M. alpine* and the same isomerase (PAI) from *P. acnes* [82]. CLA production improved to 132.6 mg/L when the additional steps were taken of overexpressing *DGA1*, optimizing culture conditions (i.e., carbon and nitrogen sources, carbon-to-nitrogen mass ratio, and CaCl_2_ content), and using glycerol as the sole carbon source [82].

### 4.2. Cyclopropane Fatty Acids

Easily converted to methylated FAs, cyclopropane fatty acids (CFAs) have applications in the lubrication and oleochemical industries because of their long-term resistance to oxidization and their fluidity at low temperatures [83,84,85]. CFAs are naturally produced in a variety of different organisms, ranging from bacteria to plants, and can represent varying percentages of total FAs (as high as 46%) [86]. During natural production processes, CFAs typically accumulate in membrane-bound phospholipids. Consequently, strategies were developed for *Y. lipolytica* to produce CFAs in the form of TAGs, which serve as a more desirable production pool [86,87].

CFA synthase from *E. coli* was overexpressed in a *Y. lipolytica* strain that had been engineered to accumulate large amounts of lipids by disrupting β-oxidation and overexpressing *DGA*1; using flask cultivation, 190 mg/L of C19CP (C19:0 cyclopropane) was produced. When fed-batch fermentation was employed, C19CP titers reached 3.03 g/L (representing 32.7% of total FAs) [86].

Alternative research took the approach of assessing how 10 CFA synthase-coding genes from different organisms affected CFA production in *Y. lipolytica* [87]. However, instead of employing the technique described above [86], the objective was to test different hybrid promoters with a view to finding the optimal promoter for producing CFAs. In the final strain, TAG degradation and remobilization were blocked, and CFA synthase from *E. coli* was overexpressed under the strong Hp8d promoter; this strain produced 2.32 g/L of CFAs (representing 45.8% of total FAs) [87]. Imatoukene and colleagues introduced *E. coli* CFA synthase into *Y. lipolytica* which was metabolically engineered to accumulate high amount of fatty acid and phospholipid remodeling [88]. The optimization of bioprocess strategy resulted in 70% (g/g DCW) of lipid accumulation, 7.49 g/L of CFA, and with a CFA productivity of 103.3 mg/L/h.

### 4.3. Ricinoleic Acid

Ricinoleic acid (RA) is a hydroxylated FA (12-hydroxy-octadeca-*cis*-9-enoic acid, C18:1-OH) that can be used as a substrate for double-bond and hydroxyl-group reactions or as an intermediate compound in reactions generating plasticizers, lubricants, dyes, inks, soaps, pharmaceuticals, food additives, cosmetics, and biofuels, among other products [89,90,91]. Recombinant production of RA was attempted using plants, but the resulting RA levels were lower than those seen in a native producer—castor seeds [91,92,93,94]. Microbial production of RA was explored using *S. cerevisiae*, *Schizosaccharomyces pombe*, and *Pichia pastoris* [92,95,96,97]. When *FAH12* (∆12-oleate hydroxylase) from *C. purpurea* was overexpressed in *S. pombe*, RA titers reached 137.4 mg/L (representing 52.6% of total FAs) [95]. To reduce the toxicity of RA, intracellular phospholipase (*PTL2*) was overexpressed, which increased RA secretion and intracellular levels 1.2 and 1.3-fold, respectively [96]. In *P. pastoris*, co-expression of *CpFAH* and *CpDGAT1* from *C. purpurea* resulted RA titers of 495 mg/L [97].

A *Y. lipolytica* strain was engineered in multiple ways to accumulate large amounts of RA (up to 43% of total lipids; more than 60 mg/g DCW) [98]. To provide the substrate for RA production, β-oxidation was blocked, and the *FAD2* gene was deleted. By deleting three DAG acyltransferases and overexpressing *LRO1*, TAGs were exclusively synthesized through the phospholipid pathway. When the strain also overexpressed *FAH12* from *C. purpurea*, RA titers reached 12 g/L (representing 60% of total lipids) in a bioreactor. Recently, secondary structure prediction and mutagenesis helped identify the crucial domain involved in CpFAH12 hydroxylation in *Y. lipolytica* [99] and a consolidated RA production process was developed by introducing RA synthesis in a strain engineered for cellulose utilization [100].

### 4.4. Polyunsaturated Fatty Acids

Polyunsaturated fatty acids (PUFAs) have attracted scientific attention because of their numerous health benefits. Of particular note are eicosapentaenoic acid (EPA, 20:5, *n*-3) and docosahexaenoic acid (DHA, 22:6, *n*-3), which are also known as long-chain omega-3 FAs. They reduce the incidence of cardiovascular diseases and prevent myocardial infarction, bronchial asthma, inflammatory bowel disease, severe depression, and several types of cancer [101]. Fish oils are the natural source of omega-3 FAs, but concerns about overfishing and pollution in marine ecosystems have led to researchers to attempt to microbially produce EPA and DHA [102,103]. A few products generated by microalgae (*Crypthecodinium cohnii* and *Schizochytrium* species) are commercially available [103,104,105]. EPA has been successfully produced in *Y. lipolytica* via a metabolic engineering strategy that introduced Δ6 desaturase, C18/20 elongase, Δ5 desaturase, and Δ17 desaturase. The strain was further engineered to overexpress an elongase from *M. alpina* and a Δ12 desaturase from *Fusarium moniliforme*, resulting in high levels of EPA (40% of total lipids) [101,104,105]. EPA production was improved even more by eliminating a competitive pathway that generated byproducts and by introducing multiple copies of genes encoding crucial enzymes (described above); the final strain produced levels of EPA that accounted for approximately 21.3% of DCW and 57% of FAMEs [106]. This strategy has led to commercial products—New Harvest™ EPA oil and Verlasso^®^ salmon—and illustrates the successful use of *Y. lipolytica* as a chassis for biotechnological applications [3]. Recently, transgenic *Y. lipolytica* strains were created that produce specific PUFAs, such as arachidonic acid (AA, 20:4, *n*-6), EPA, docosapentaenoic acid (DPA, 22:5, *n*-3), and DHA [107]. The multifunctional polyketide synthase (PKS)-like PUFA synthases from *Aetherobacter fasciculatus* and *Minicystis rosea* were introduced in a diverse hybrid form to *Y. lipolytica* and have been employed to produce mainly DHA and AA, respectively. Under conditions of improved fermentation, the engineered strain had DHA titers of 350 mg/L (representing 16.8% of total FAs).

### 4.5. Emerging Target Compounds 

#### 4.5.1. Acetylated Triacylglycerols

Structurally, 3-acetyl-1,2-diacylglycerols (acTAGs) are similar to regular TAGs. However, the two groups differ at the *sn*-3 position, which is esterified by an acetate rather than by an FA [108]. The acTAGs are naturally found in plants of the family Celastraceae and in animals like *Cervus nippon* and *Eurosta solidaginis*. Because of their acetyl moiety, acTAGs have lower viscosity and calorific value than TAGs, which are characteristics of value in certain industrial products, like biodiesels, emulsifiers, lubricants, and plasticizers. When heterologous diacylglycerol acetyltransferase (DAcT) from *Eunonymus europaeus* was introduced into a *Y. lipolytica* WT strain, acTAGs represented about 20% of total lipids [108]; when *DAcT* was overexpressed in a non-TAG-accumulating strain, acTAGs (representing 10% of total lipids) were the only stored lipid.

#### 4.5.2. Methylated Fatty Acids

Saturated branched (methyl) lipids like 10-methylstearic acid have desirable properties, such as fluidity at low temperatures and oxidative stability. These characteristics are useful when the compounds are employed in lubricants and specialty fluids. Oleic acid and methionine can serve as substrates that are converted into 10-methylenestearic acid by methyltransferase (tmsB) and then further transformed into 10-methylstearic acid by reductase (tmsA). A relatively recent study explored the effects of co-expressing *tmsB* and *tmsA* of different origins in several microorganisms, including *Y. lipolytica* [109]. It was found that the co-expression of *tmsA* and *tmsB* from *Thermomonospora curvata* could produce branched 10-methyl and 10-methylene FAs in *Y. lipolytica*. Fusion enzymes (tmsA-B), which displayed the activities of both enzymes, allowed 10-methylstearic acid to be produced (at levels representing more than 20% of total FAs).

#### 4.5.3. Pheromones (Fatty Alcohols)

In pest control efforts, insect sex pheromones can be used as an alternative to insecticides, which have adverse effects on human health and the environment. Consequently, the biotechnological production of pheromones has recently begun to draw interest.

The sex pheromones of moth pests, (Z)-hexadec-11-en-1-ol (Z11-16OH) and (Z)-tetradec-9-en-1-ol (Z9-14OH), have been produced in *Y. lipolytica* by introducing a desaturase from *Amyelois transitella* (*AtrD11*) and a reductase from *Helicoverpa armigera* (*HarFAR*) [110]. To improve pheromone production, fatty alcohol degradation was inhibited (*hfd1-4Δ fao1Δ*), and the size of the precursor pool was boosted by inhibiting FA degradation and storage (*pex10Δ*, truncated pGPAT). The final strain contained multiple copies of the genes encoding the desaturase and reductase. When grown in a bioreactor, it produced Z11-16OH titers of 2.6 g/L. The efficiency and specificity of these pheromones were verified by successfully trapping male *H. armigera* moths in a cotton field.

Bumble bee pheromones were synthesized in *Y. lipolytica* by overexpressing fatty acyl-CoA reductases: *BlapFAR4* from *Bombus lapidaries* and *BlucFAR1* from *Bombus lucorum* [111]. Each enzyme displayed a different substrate preference. *BlapFAR4* overexpression produced mostly C16:0-OH and C16:1∆^9^-OH, while *BlucFAR1* overexpression produced a mixture of saturated fatty alcohols (C18–C24). The highest fatty alcohol titers (166.6 mg/L) were obtained by overexpressing *BlucFAR1* in a strain that accumulated large amounts of lipids.

## 5. Conclusions and Perspectives

*Y. lipolytica* is a promising host for producing lipid-based chemicals. Great strides have been made in using this non-conventional yeast, thanks to extensive research into lipid metabolism and the development of metabolic engineering tools. The deployment of genetic tools and metabolic models has further accelerated improvements in oleochemical production in this yeast. Unusual lipids are targets of particular interest because of their diverse applications. Native producers of unusual lipids are rare, making large-scale production challenging. Consequently, using microbes as factories for generating unusual lipids should be a more economically feasible and environmentally sustainable approach. This review discussed the diverse unusual lipids that have been produced in *Y. lipolytica* via metabolic engineering. However, most of the research described involves production at the laboratory scale or proof-of-concept studies. It is hoped that combining metabolic engineering strategies and systems biology will help identify the rate-limiting steps present in the processes used to generate unusual lipids, thus boosting their production. The range of potential unusual lipids can be broadened by identifying novel enzymes via system metabolic engineering. Additionally, to permit production at industrial scales, it will be necessary to optimize fermentation processes, maximize yield, and reduce the formation of byproducts. Production costs can be reduced by utilizing cheap raw materials, a topic that has already been extensively studied in *Y. lipolytica*. By addressing this suite of issues, the microbial production of unusual lipids will move much closer to becoming commercially viable.

**Table 1:** The production of unusual lipids via metabolic engineering in *Y. lipolytica*.

Product	Strain Genotype	Strategy	Production Details	% of Total Lipids	Reference
MCFA	po1f *pTEF-UcACPT*	Introduction of heterologous acyl-ACP thioesterases	C8-FA	14	[54]
MCFA	po1f *pTEF-CpaACPT*	Introduction of heterologous acyl-ACP thioesterases	C10-FA	57	[54]
MCFA	po1g *hFAS-EcTesA ′^1^*	Introduction of heterologous acyl-ACP thioesterases and hybrid fatty acid synthase-thioesterase	C14-FA: 29.2% of DCW		[25]
MCFA	po1d *pox1-6∆ fas1-I1220W*	Engineering of native FAS system	C14-FA: 2.02% of DCW	11.6	[55]
MCFA	po1d *pox1-6∆ elo1∆ fas1-I1220W pTEF-egDGAT*	Inhibition of native elongation; introduction of heterologous diacylglycerol acyltransferase	C14-FA: 1.25 g/L	45	[56]
OCFA	po1d *phd1Δ mfe1∆ tgl4Δ pTEF-DGA2 pTEF-GPD1*	Inhibition of precursor catabolism (propionyl-CoA); high levels of lipid accumulation	0.57 g/L	41.9	[66]
OCFA	po1d *phd1Δ mfe1∆ tgl4Δ pTEF-DGA2 pTEF-GPD1 hp4d-LDP1 pTEF-Repct pTEF-RebktB*	Increase in precursor pool size; high levels of lipid accumulation ^2^	1.87 g/L	62.1	[67]
OCFA	po1d *pox1-6*∆ *tgl4Δ* pTEF-*DGA2* pTEF-*GPD1 pTEF-AAT2 pTEF-THR1 pTEF-THR4 pTEF-ILV1 pTEF-HOM3 pTEF-HOM2 pTEF-HOM6*	Overexpression of threonine synthesis pathway; high levels of lipid accumulation ^2^	0.36 g/L	5.6	[68]
VLCFA	po1f *pex10Δ F1::UT-MaELO3 UT-AtKCS UT-CraKCS UT-EcAldh*	Introduction of heterologous β-ketoacyl-CoA synthases and heterologous elongase; inhibition of peroxiome biogenesis; increase in precursor pool size	280 mg/L		[74]
VLCFA	po1d *fad2Δ pTEF-FAE1*	Inhibition of Δ12 desaturation; introduction of heterologous elongase	C22:1-FA: 887 mg/L	9	[73]
CLA	polh *(hp4d-oPAI)* × *24*	Introduction of heterologous CLA-producing isomerase with large numbers of gene copies		5.9	[79]
CLA	po1h (hp16d-oPAI) × 8 hp4d-MaFAD2	Introduction of heterologous CLA-producing isomerase and desaturase	30% of DCW	44	[80]
CLA	po1d *pox1-6Δ dga1Δ dga2Δ lro1Δ fad2Δ pTEF-FAD2 pTEF-oPAI-LEU2ex pTEF-oPAI-URA3ex*	Introduction of heterologous CLA-producing isomerase; overexpression of *FAD2*; high levels of lipid accumulation ^2^	302 mg/L	6.5	[81]
CLA	po1h *hp4d-PAI hp4d-MA12D hp4d-DGA1*	Introduction of heterologous desaturase, dicylglycerol acyltransferase, and isomerase	132.6 mg/L	5.2	[82]
CFA	po1f *pex10Δ mfe1Δ UAS1B16-TEF-DGA1 (UAS1B16-TEF-ycoCFA)* × *2*	Introduction of heterologous CFA synthase; high levels of lipid accumulation ^2^	C19CP: 3.13 g/L	32.7	[86]
CFA	po1d *pox1–6Δ tgl4Δ Hp8d-CFAs*	Introduction of heterologous CFA synthase; high levels of lipid accumulation ^2^	2.3 g/L	45	[87]
CFA	po1d *pox1-6Δ tgl4Δ pTEF-GPD1 pTEF-DGA2 hp8d-CFA pTEF-LRO1*	Introduction of heterologous CFA synthase; overexpression of native LRO1; optimization of carbon source	7.5 g/L	19.6	[88]
RA	po1d *pox1-6Δ dga1Δ lro1Δ dga2Δ fad2ΔpTEF-CpFAH12 (pTEF-CpFAH12)* × *2 pTEF-LRO1*	Introduction of heterologous fatty acid hydroxylase; increase in precursor pool size	12 g/L	60	[98]
PUFA	*ATCC 20362 (Δ12 DES)* × *3 (Δ6 DES)* × *2 (C18/20 ELO)* × *4 (Δ5 DES) × 5 (Δ17 DES)* × *3 (C16/18 ELO)* × *2*	Introduction of desaturases and elongases; push and pull of carbon into the engineered pathway	EPA	40	[101]
PUFA		Introduction of desaturases and elongases with multiple gene copies; inhibition of the competitive pathway	EPA:21.3% of DCW	57	[106]
PUFA	Po1h *hp4d-Pfa hp4d-PptAf4*	Introduction of multifunctional polyketide synthase (PKS)-like PUFA synthases from *Aetherobacter fasciculatus* and *Minicystis rosea*	DHA: 350 mg/L	16.8	[107]
acTAG	Po1d *pTEF-EeDAcT*	Introduction of heterologous diacylglycerol acetylransferase	4.06% of DCW	20	[108]
meTAG		Introduction of heterologous methyltransferase and reductase	10-methylstearic acid	20	[109]
Pheromones	Y-17536 *ku70Δ Cas9 hfd1Δ hfd4Δ pex10Δ fao1Δ PGPAT100bpPr (AtrΔ11)x3 HsuFAR (HarFAR)* × *2*	Introduction of heterologous desaturase and reductase with multiple gene copies	Z11-16OH: 2.57 g/L		[110]
Pheromones	po1d *Δpox1-6 Δtgl4+pXPR2-SUC2 pTEF-DGA2 pTEF-GPD1 8UAS-pTEF-BlucFAR1*	Introduction of heterologous fatty acyl-CoA reductases	166.6 mg/L		[111]

^1^ hybrid protein of the truncated FAS1 with truncated EcTesA. ^2^ high levels of lipid accumulation: metabolic engineering strategies used to improve total lipid accumulation as described in Section 2.2. The detailed strategies for each strain can be found in Section 3 and Section 4 of the text.

## Abbreviations

ACPT	acyl-ACP thioesterase
AcTAGs	acetylated TAGs
CFA	cyclopropane fatty acid
CLA	conjugated linoleic acid
C/N ratio	carbon-to-nitrogen ratio
DacT	diacylglycerol acetyltransferase
DAG	diacylglycerol
DCW	dry cell weight
DHA	docosahexaenoic acid
DPA	docosapentaenoic acid
ECFA	even-chain fatty acid
EPA	eicosapentaenoic acid
ER	endoplasmic reticulum
FA	fatty acid
FAEE	fatty acid ethyl ester
FAME	fatty acid methyl ester
FAS	fatty acid synthase
FFA	free fatty acid
KAS	beta-ketoacyl-ACP synthase
KS	ketoacyl synthase
LA	linoleic acid
LB	lipid body
LCFA	long-chain fatty acid
LPA	lysophosphatidic acid
MAG	monoacylglycerol
MCFA	medium-chain fatty acid
MFE	multifunctional enzyme
MPT	malonyl/palmitoyl transacylase
OCFA	odd-chain fatty acids
PA	phosphatidic acid
PCT	propionyl-CoA transferase
PKT	polyketide synthase
PPP	pentose phosphate pathway
PUFA	polyunsaturated fatty acid
RA	ricinoleic acid
SCFA	short-chain fatty acid
TAG	triacylglycerol
TALEN	transcription activator-like effector nuclease
VLCFA	very-long-chain fatty acid
WT	wild type
